# Precision Treatment of Metachronous Multiple Primary Malignancies Based on Constructing Patient Tumor-Derived Organoids

**DOI:** 10.3390/biomedicines12122708

**Published:** 2024-11-27

**Authors:** Yicheng Wang, Haotian Chen, Zhijin Zhang, Yanyan He, Ji Liu, Baoshuang Zhao, Qinwan Wang, Jiangmei Xu, Shiyu Mao, Wentao Zhang, Xudong Yao, Wei Li

**Affiliations:** 1 Department of Urology, Shanghai Tenth People’s Hospital, School of Medicine, Tongji University, Shanghai 200072, China; 2050700@tongji.edu.cn (Y.W.); chenhaotian9710@163.com (H.C.); 2231200@tongji.edu.cn (Z.Z.); 13661483022@163.com (Y.H.); drliu7087797@163.com (J.L.); aiyu5410@163.com (J.X.); maoshiyu1144@sina.com (S.M.); yaoxudong1967@163.com (X.Y.); 2 School of Medicine, Tongji University, Shanghai 200092, China; 3 Urologic Cancer Institute, School of Medicine, Tongji University, Shanghai 200435, China; 4 Department of Pathology, Shanghai Tenth People’s Hospital, School of Medicine, Tongji University, Shanghai 200072, China; 5 Department of Hepatobiliary Surgery, Yiwu Central Hospital, Yiwu 322000, China; baidubuqin0302@sina.com; 6 Department of Central Laboratory, Clinical Medicine Scientific and Technical Innovation Park, Shanghai Tenth People’s Hospital, Shanghai 200435, China; qinwan_yiyi2011@163.com

**Keywords:** multiple primary malignant neoplasms (MPMNs), patient-derived organoids, precision medicine

## Abstract

When a patient has two or more primary tumors, excluding the possibility of diffuse, recurrent, or metastatic, they can be defined as having multiple primary malignant neoplasms (MPMNs). Moreover, cases of three primary urinary tract tumors are very rare. Here, we reported a patient of MPMNs with four primary tumors, including three urinary tract cancers (renal cancer, prostate cancer, and bladder cancer) and lung cancer. The four tumors appeared over 13 years, and pathological results confirmed that they were all primary tumors after different surgeries. In addition, we established patient-derived organoids (PDOs) by collecting tumor specimens. Hematoxylin-eosin (H&E) staining of PDOs showed that the organoids were histopathological consistent with parental tumor. Immunohistochemistry showed that PDOs can also reflect the expression of pathological markers in patients. At the same time, PDOs may also serve as “avatars” of patients to predict sensitivity to different drugs. In summary, we reported a case of MPMNs with four primary tumors and established PDOs from its tumor specimens. A personalized treatment strategy was established based on the histopathological characteristics of the organoids.

## 1. Introduction

With the advancement of diagnostic techniques, an increasing number of tumors can be detected at an early stage. Although the incidence of multiple primary malignant neoplasms (MPMNs) is on the rise, they remain rare in clinical practice [1]. The concept of multiple primary tumors was first proposed by Billroth in 1889 [2]. In 1932, Warren and Gates established the diagnostic criteria of MPMNs, which included the presence of two or more malignant primary neoplasms and excluded diffuse, recurrent, and metastatic tumors [3]. The International Agency for Research on Cancer (IARC) defines synchronous MPMNs (SMPMNs) as tumors occurring at different sites diagnosed at intervals of less than 6 months, and metachronous MPMNs (MMPMNs) as those with an interval of more than 6 months [4]. Currently, the reported incidence of MPMNs in the literature varies greatly, ranging from 1% to 17% abroad [5]. In China, there are only a few hospital-based single-center studies available, with an overall incidence rate of 0.4% to 2.0% [6]. Despite these variations, the increasing number of clinical reports of MPMNs indicates that patients with four primary tumors are still a rare occurrence. MPMNs cases are predominantly double primary (around 90%), with triple primary, quadruple primary, and quintuple primary and above accounting for around 5%, 3% and 1% [7], respectively. This study reports a patient with MPMNs who had four different tumors, three of which originated from the urinary tract, a clinically rare occurrence.

Providing safe and effective personalized treatment for patients with MPMNs remains a challenge due to their rarity and specificity. Organoids could be a valuable tool in addressing this issue. Patient-derived organoids (PDOs) have emerged as a powerful pre-clinical model with the potential to direct clinical personalized treatment [8]. In recent years, it has become possible to culture organoids from a wide range of tumor tissues [9]. PDOs accurately replicate the structural features of tumors, which can reflect the histopathology and molecular diversity of the original tumor [10]. Studies have demonstrated that organoids maintain characteristics of the original tumor samples, including phenotype, genetic diversity, and mutational characteristics [11]. In addition, PDOs can replicate the heterogeneity of tumors that is not present in cell lines, by reflecting the interactions between the tumor and the surrounding cells or stroma [10]. PDOs have significant potential in identifying therapeutic targets and verifying drug responses [12], thereby providing new ideas for clinical precision diagnosis and treatment. Organoids can also be employed for drug screening by correlating the genetic background of a tumor with drug response [13]. Drug screening using organoids is a rapid, high-throughput, and clinically relevant method [14]. Current studies have shown that most PDOs exhibit a therapeutic response that is consistent with the patient’s initial response to therapy [15]. Further, PDOs are sensitive to cytotoxic drugs, which enables better prediction of clinical response after use in patients [16]. The use of PDOs to predict drug response in vitro prior to treatment is intended to provide better treatments for patients and predict treatment outcomes. Therefore, the culture of organoids warrants further clinical studies due to its significance in studying the primitive tumor cells in patients with MPMNs, exploring their pathogenesis, and proposing precise therapeutic regimens.

This study describes a rare case of a 73-year-old male patient who suffered from MPMNs of the lung, bladder, prostate, and kidney. Organoids were established by isolating the patient’s renal cancer specimens to simulate the real tumor tissues and to inform the subsequent treatment.

## 2. Materials and Methods

### 2.1. Organoids

Renal cancer tissues were acquired via laparoscopic partial nephrectomy. The tissue was sliced into sections approximately 1–2 mm thick, then digested for an hour using type IV collagenase (1 mg/mL, C9891, Sigma-Aldrich, Saint Louis, MO, USA) and Y-27632 (HY10071, 10 μM MCE, Shanghai, China). The suspension was passed through a 70 μm mesh filter, and the collected precipitate was suspended in 200 μL of basement membrane extract (BME, 3533-001-02, R&D, Waltham, MA, USA), before being seeded into pre-heated 12-well plates. Once the BME solidified, human renal cancer organoid culture medium was added for cultivation [17].

### 2.2. H&E Staining and IHC Staining

Tissues and organoids were subjected to routine staining protocols for hematoxylin-eosin staining and immunohistochemical (IHC) staining. Specimens were preserved in cold 4% ParaFormAldehyde (PFA) and then encased in paraffin blocks. Following paraffin removal, dehydration, antigen recovery, and blocking, sections were incubated overnight at 4 °C with antibodies. Subsequently, all sections were treated with biotinylated goat anti-rabbit IgG for 20 min at room temperature, followed by a 30 min incubation with streptavidin-horseradish peroxidase. In the final step, diaminobenzidine-H_2_O_2_ and hematoxylin (C0107, Beyotime, Shanghai, China) were employed for staining the tissues. Immunohistochemistry for ccRCC markers (CAIX(ab243660), RCC(ab277482), CD10(ab256494), Abcam, Waltham, MA, USA), vascular markers (VEGF, ab1316, Abcam, Waltham, MA, USA) and immunotherapy markers (PD-L1, ab205921, Abcam, Waltham, MA, USA) were performed.

## 3. Results

### 3.1. Case Presentation

A male patient presented to our hospital in July 2023 with dysuria and increased urinary frequency that had persisted for three years without any identifiable triggers. While his symptoms improved with medications like Finasteride, a follow-up examination revealed an elevated PSA level of 97.5 ng/dL. He was subsequently admitted for further investigation and management of suspected prostatic hyperplasia and elevated PSA.

Detailed medical history revealed an episode of hematuria twelve years prior. A urinary ultrasound at that time identified a 24 × 15 mm bladder mass, leading to a transurethral resection of the bladder tumor (TURBT) on 2011. Postoperative pathology confirmed papillary uroepithelial carcinoma of the bladder (pTaN0M0), with immunohistochemistry positive for p53, HER-2, PCNA, CK, 34βE12, and p63. No recurrence of bladder cancer was observed in the following years.

Additionally, the patient underwent a right middle lung lobectomy and lymph node dissection at another hospital in November 2016. Pathological analysis revealed papillary invasive adenocarcinoma of the right middle lobe lateral segment (pT1N0M0) with vesicular and volvulus-like components, measuring 0.8 × 0.5 × 0.5 cm. Immunohistochemistry showed EGFR positivity with exon 21 L858R mutation, while ALK and K-ras were negative. No recurrence has been observed since the lung surgery.

**Figure 1:**
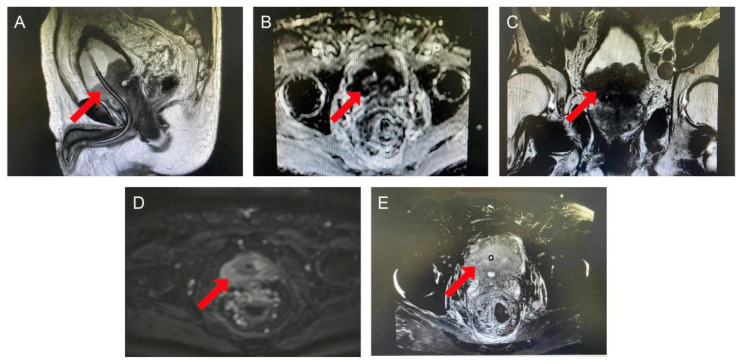
MRI images of the prostate. The figure indicates a mass in the prostate, involving the posterior wall of the bladder, bilateral seminal vesicles, and the anterior wall of the distal rectum. Lymph nodes in the pelvic wall and inguinal area were discovered to be enlarged, accompanied by numerous abnormal signal spots in the pelvis. (**A**–**C**) T2WI images of prostate cancer in sagittal, axial, and coronal views. There is a lesion in the prostate, which appears as a low signal. (**D**) DWI images of prostate cancer, with the tumor exhibiting a high signal. (**E**) ADC images of prostate cancer, indicating a lower ADC value for the tumor. The red arrow points to the prostate cancer.

During this hospitalization, the patient underwent a magnetic resonance imaging (MRI) scan, and the results revealed that the patient had an abnormality in the prostate involving the posterior wall of the bladder, bilateral seminal vesicles and the distal anterior wall of the rectum, which was considered a malignant neoplastic lesion and required further puncture pathology (Figure 1A–E). Enlarged lymph nodes were found in the pelvic wall and inguinal region, along with multiple foci of abnormal signal in the pelvis, indicating the possibility of metastasis. Based on the patient’s medical history and relevant investigations, a diagnosis of prostate malignancy (cT4N1M1) was considered. To further clarify the diagnosis, the patient underwent an ultrasound-guided prostate biopsy, and the pathological results diagnosed prostate adenocarcinoma with a Gleason score 9 (5 + 4). Immunohistochemical staining of the tumor cells showed PSA (+), PSMA (+), P504S (+), PTEN (+), c-Myc (30%+), CerbB2 (1+), MSH2 (+), PD-L1 (<5%+). 

**Figure 2:**
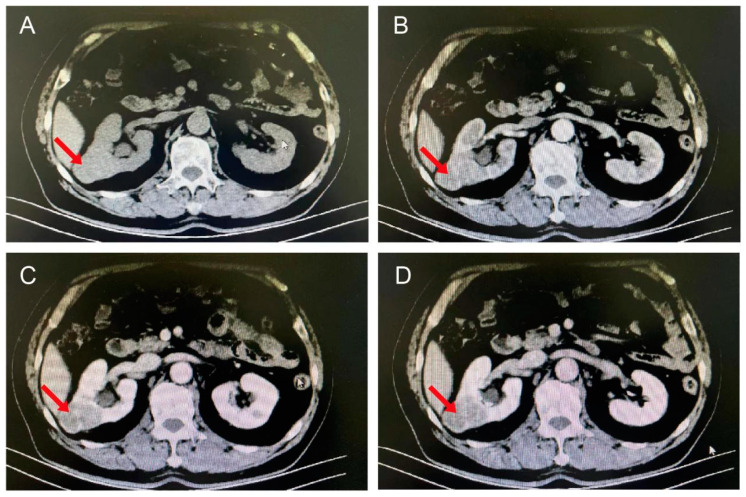
Enhanced CT images of the kidneys. The figure indicates a 4.2 × 3.5 × 4 cm nodule in the middle part of the right kidney, with dilatation and fluid accumulation in the right renal pelvis, calyces, and the upper segment of the ureter. (**A**) Unenhanced. A solitary lobulated mass is present within the right kidney parenchyma, protruding beyond the renal contour. The mass has uneven density, with irregular low-density areas inside and unclear margins. (**B**) Enhanced arterial phase. The mass shows obvious non-homogeneous enhancement, with its density close to that of the renal cortex, and a non-enhanced necrotic area visible in the center. (**C**,**D**) Enhanced portal venous phase and enhanced venous phase. The mass demonstrates rapid delineation, with a density lower than that of the normal renal parenchyma. The red arrow points to the kidney cancer.

In addition, computed tomography (CT) of the patient’s lungs showed that the patient had a nodule in the middle level of the right kidney. To further clarify the diagnosis, a renal enhancement CT was performed. The results showed a 4.2 × 3.5 × 4 cm mass in the middle of the right kidney, with possible multiple retroperitoneal lymph node metastases (Figure 2A–D). According to the patient’s medical history and the above examinations, a renal malignant tumor (cT1N1M0) was diagnosed.

**Figure 3:**
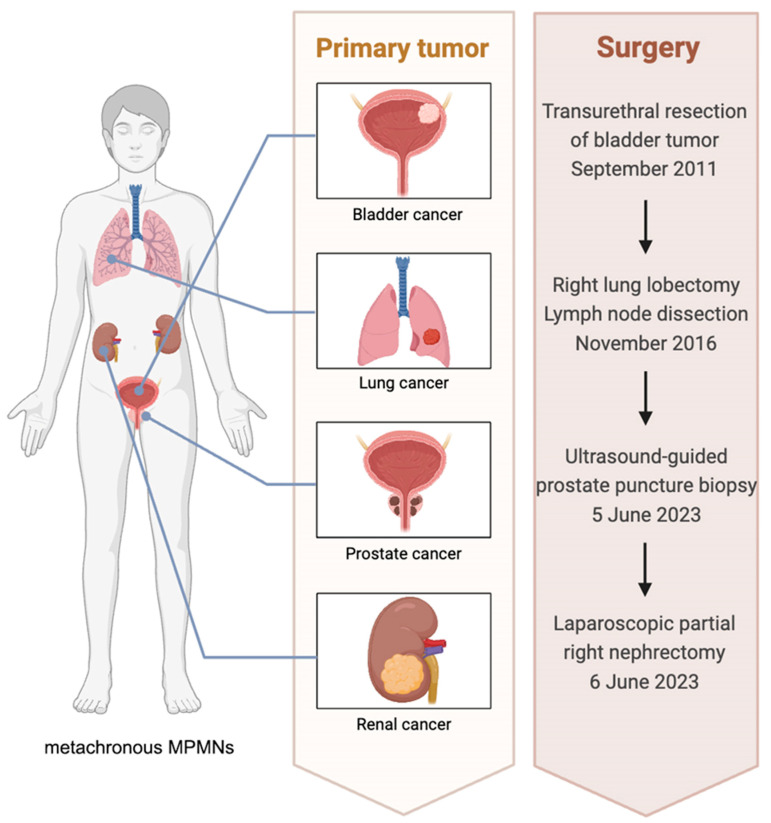
Progression and corresponding surgeries of four primary tumors in the patient. The patient received a transurethral resection of bladder tumor to treat bladder cancer in September 2011. A right lobectomy and lymph node dissection for lung cancer were conducted in November 2016. Prostate cancer was diagnosed on 5 June 2023 through an ultrasound-guided prostate biopsy. A laparoscopic partial resection of the right kidney for kidney cancer treatment was performed on 6 June 2023.

As the patient’s prostate cancer was classified as cT4N1M1, surgical treatment was not recommended. Therefore, we first performed a laparoscopic partial nephrectomy for the treatment of the patient’s kidney tumor. Postoperative pathological results indicated clear cell renal cell carcinoma (grade 3). Immunohistochemical staining showed that the tumor cells were positive for CK-p, Vimentin, PAX8, CAIX, P504S, Claudin7, FH, a small amount of positive RCC, Ki-67 (20%+), P53 (10%+), and TFE-3 (weakly positive). Following surgery for renal cancer, the patient underwent endocrine therapy for prostate cancer with Leuprorelin Acetate and Apalutamide (Figure 3).

Four primary tumors were comprehensively diagnosed including bladder malignancies, lung adenocarcinoma, prostate adenocarcinoma and clear cell renal cell carcinoma, based on medical history and the confirmation of two primary tumors during this admission. Here, we have summarized the clinical information of the patient’s entire diagnostic and treatment process by compiling a table (Table 1).

### 3.2. Established ccRCC Organoid

**Figure 4:**
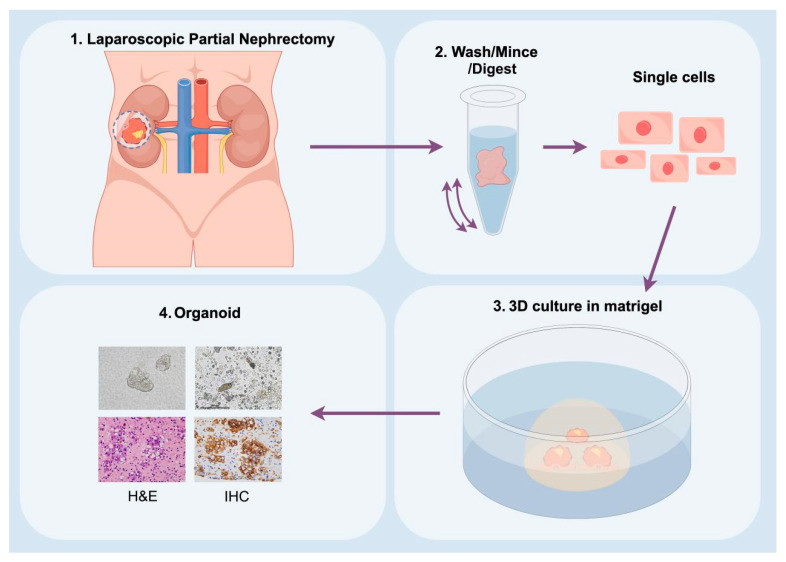
Graphical illustration of the organoid construction and prediction of drug targets. 1. The renal tumor specimens are obtained through laparoscopic partial nephrectomy. 2. Washing, slicing, and digestion to isolate tumor cells. 3. 3D cultured in Matrigel. 4. H&E and IHC staining are performed on the constructed organoids to predict drug targets.

**Figure 5:**
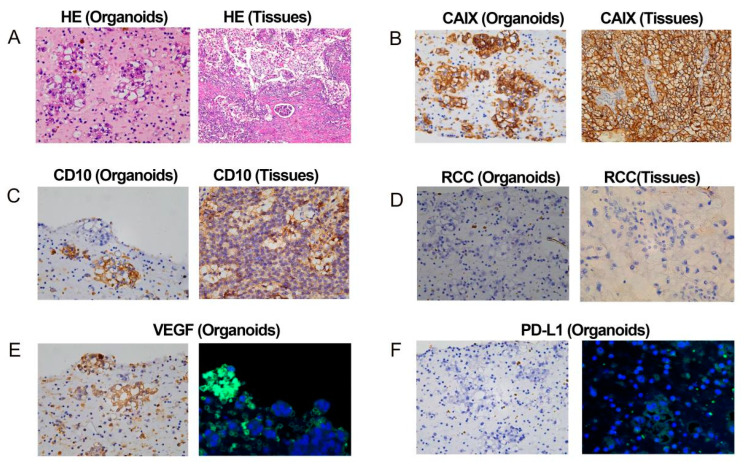
The organoids preserve the histopathological features inherent to the original tumor tissues. (**A**) Representative H&E stainning images of primary ccRCC tumors and organoids. (**B**) Representative IHC staining images of original tumors and organoids for CAIX. (**C**) Representative IHC staining images of original tumors and organoids for CD10. (**D**) Representative IHC staining images of original tumors and organoids for RCC. (**E**) Representative IHC staining images of original tumors for VEGF, and organoids stained VEGF by immunofluorescence (Green). (**F**) Representative IHC staining images of organoids for PD-L1, and organoids stained PD-L1 by immunofluorescence (Green).

The patient’s renal cancer tissue samples were isolated for the establishment of organoids (Figure 4), and the histopathological characteristics of both tumor tissues and organoids were observed using H&E staining and immunohistochemistry. The H&E staining of the organoids reveals large tumor cells with round or polygonal shapes, abundant clear cytoplasm, enlarged nuclei and mild to moderate nuclear pleomorphism. Additionally, the tumor cells are arranged in nests, with a rich capillary and sinusoidal network in the stroma (Figure 5A). Immunohistochemistry was also used to verify similarity between the organoid and parental tumor. It is evident that in both the patient’s original tumor tissues and the organoids, CAIX, RCC are positively stained. This demonstrated that the organoids can preserve the histopathological features inherent to the original tumor tissues (Figure 5B,D).

Given that organoids retain the heterogeneity of the original tumor, they can be used in conjunction with immunohistochemistry to clear diagnosis and identify potential therapeutic targets. The expression of CD10 in the diagnosis of clear cell renal cell carcinoma allows for differentiation from other renal tumor types (like chromophobe cell carcinomas) (Figure 5C) [18]. The positive VEGF staining in organoids indicates that the tumor has significant angiogenic activity, providing a rationale for choosing targeted therapy drugs (Figure 5E). The positive PD-L1 staining in organoids is critically instructive in the selection of immunotherapy strategies (Figure 5F).

## 4. Discussion

Simultaneous tumors and metachronous tumors were classified according to whether the diagnostic interval between tumors was more than six months [19]. In our case study, the patient developed four primary malignant tumors in two organ systems (urinary and respiratory) of different pathological types, meeting the diagnostic criteria for MPMNs [20]. The patient had a 5-year interval between the onset of bladder cancer and lung cancer, and a 7-year interval from lung cancer to the detection of renal and prostate cancers in this visit, aligning with the progression of metachronous MPMNs. According to the literature, the incidence of four primary malignant tumors is approximately 3% [7]. However, cases of patients suffering from three tumors originating in the urinary tract (bladder, kidney and prostate) are rarely reported.

Due to the large heterogeneity of MPMNs and the limited clinical evidence available, high-quality research findings to guide the treatment of MPMNs are lacking. The China Anti-Cancer Association’s guideline for diagnosis and treatment of cancer of multiple and unknown primaries (2023 edition) recommends that the treatment of multiple primary malignant tumors should be based on the principles of treatment for each primary tumor. For metachronous MPMNs, the stage of the primary tumor needs to be adequately evaluated. The choice of surgical, medical or radiological treatment should be made taking into account the stage, the degree of malignancy and the tolerance of organ function [7]. The patient underwent TURBT for papillary uroepithelial carcinoma of the bladder (pTaN0M0) and right lung lobectomy and lymph node dissection for papillary invasive adenocarcinoma of the lung (pT1N0M0). Additionally, the patient underwent laparoscopic partial nephrectomy for clear cell renal cell carcinoma (cT1N1M0) following admission. Unfortunately, surgical treatment is generally not recommended for the patient’s stage IV B prostate adenocarcinoma (cT4N1M1). Apalutamide is a new generation of non-steroidal AR inhibitors, whose binding to the androgen receptor is 7 to 10 times higher than that of traditional anti-androgen receptor drugs [21]. It can effectively inhibit the function of androgen receptor, reduce its DNA binding efficiency and nuclear translocation, thus achieving the effect of inhibiting the proliferation of prostate tumor cells [22]. Treatment with Apalutamide significantly prolongs the median progression-free survival and reduces the risk of distant metastasis or death [23]. Therefore, we chose to treat our patients with Apalutamide.

With the development of genomics and the exploration of molecular biology, the treatment of tumors has stepped into the era of precision therapy. Precision medicine, which is based on molecular characteristics of tumors or their microenvironment [24], has become a new research hotspot. Tumor sequencing work is used to search for therapeutic targets, promoting the development of precision medicine and thus offering more possibilities for the effective treatment of tumor patients [25]. While DNA sequencing can provide information on driving factors of cancer [26], cancer development and progression are influenced by a wide array of factors beyond just genetic changes. DNA sequencing alone may not fully capture these other regulatory factors that contribute to the complexity of tumor biology. In addition, biomarkers that can clearly predict drug response have not yet been identified for some tumors, such as ccRCC [27,28]. In this context, the use of precision therapy based on next-generation sequencing (NGS) technology has limitations. Accordingly, the aim is to comprehensively capture multifaceted information on tumor biology by mimicking the behavior of the original tumor, which can further improve precision medicine. Patient-derived organoids are able to simulate tumors in vitro, encapsulating the histopathology and molecular diversity of the original tumor. An organoid reflects the interaction between the tumor and the surrounding cells or stroma by mimicking the tumor microenvironment [29], which possesses tumor heterogeneity and thus is better at reflecting the most realistic features of the tumor. Based on the above characteristics, organoids play an important role in predicting drug responses, exploring promising integrated treatment strategies, and discovering novel anticancer targets and promising drug candidates [30].

The occurrence and development of MPMNs have not been fully elucidated. Their high degree of heterogeneity require clinicians to individualize treatment to the patient. Organoids can be used to choose drugs, screen drugs and predict drug response, guiding us to provide more precise treatments for patients and improve their prognosis [31]. In this study, we cultured renal cancer specimens from the patient into organoids, simulating the real tumor tissues. Carbonic anhydrase IX (CAIX) exhibits elevated expression in clear cell renal cell carcinoma, serving as a diagnostic marker [32]. RCC denotes an antibody specific for renal cell carcinoma, which utilized for the accurate identification of renal cell carcinoma and its subtypes [18]. According to the H&E staining and immunohistochemical staining results of both tumor and organoid tissues, the organoids retained the histopathological features of the tumor tissues. The positive staining results for VEGF and PD-L1 suggest that the patient’s tumor may respond well to TKIs and inhibitors of the PD-1/PD-L1 pathway. Previously, researchers have utilized organoids to assess the drug sensitivity towards TKIs [33]. Immunotherapy targets immune regulation pathways to control cancer cells [34]. Present advancements in organoid technology have made it possible to retain tumor-infiltrating T lymphocytes [35], thus recapitulating the effects of immunotherapy, which enables the forecasting of immunotherapy outcomes through organoids.

However, a regret of this study is the inability to integrate the organoids’ predicted drug responsiveness with the patient’s treatment; thus, we cannot verify if the drug responses predicted by the organoids are consistent with the clinical outcomes. In the future, with the continuous establishment of biobanks and diversification of sources, organoids can be widely used as preclinical predictive models for drug screening, biomarker research and personalized treatment development. In addition, the use of organoids is also expected to provide a platform and insights for research into the mechanisms of MPMNs occurrence. Nevertheless, organoids also present certain limitations, including their inability to replicate blood perfusion, mechanical stress, and a comprehensive immune microenvironment [36]. Moreover, organoids are unable to simulate the potential connections or cellular communication between multiple primary tumors in patients with MPMNs. Microphysiology systems (MPS), also called organs-on-chips, are miniature functional units of organs constructed from multiple cell types in a variety of physical and biochemical environments, which hold promise for remedy these deficiencies [37]. Edington et al. developed a “4-way” platform that could accommodate 4 MPSs including lung, liver, gut, and endometrium [38]. This system was capable of accurate prediction of secreted liver protein profiles and maintenance of phenotypic markers for up to two weeks. In addition, microphysiological devices were constructed utilizing collagen hydrogels to investigate the influence of distinct immune cell populations on tumor cells [39,40]. It should be noted that the two technologies are not in conflict. MPS is concerned with the control and monitoring of cell function, whereas organoids are focused on simulating tissue and organ physiology. The combination of these two technologies can help to overcome their individual limitations while simultaneously enhancing their advantages [41]. It is anticipated that patient-derived MPS will emerge as novel models and powerful tools for personalized treatment of patients with MPMNs [42].

## 5. Conclusions

In summary, MPMNs exhibit significant disease heterogeneity, and the causes and mechanisms of their occurrence are not yet clear. Thus, the treatment of MPMNs patients necessitates a combination of personalized and precise therapy tailored to each patient and the characteristics of their tumors, optimizing the current treatment principles for each primary tumor. How to provide personalized treatment for MPMNs patients is a challenge that needs to be tackled in future research. Organoids can replicate the pathological features of primary tumor tissues and act as preclinical predictive models to detect potential therapeutic targets, aiming to offer precise treatment for patients. In this case, the diagnosis of four primary tumors and the establishment of organoids to predict therapeutic efficacy lay the foundation for providing precision treatment to patients with MPMNs. However, organoids are constrained in their ability to replicate blood perfusion, mechanical stress, and a fully integrated immune microenvironment, which prevents them from effectively simulating potential interactions or cellular communication between multiple primary tumors in MPMNs patients. In the future, integrating new technologies such as MPS with organoids holds promise as a powerful tool for the personalized treatment of MPMNs patients.

## Figures and Tables

**Table 1:** Case Presentation and Clinical Score of the Patients Before and After Treatment.

	Radiological Findings	Pathological Findings	Surgical Procedure	Therapeutic Drugs
Bladder cancer	A 24 × 15 mm bladder mass	Papillary uroepithelial carcinoma of the bladder	Transurethral resection of the bladder tumor	/
Lung cancer	/	Papillary invasive adenocarcinoma of the right middle lobe lateral segment	Right middle lung lobectomy and lymph node dissection	/
Prostate cancer	An abnormality was found in the prostate involving the posterior wall of the bladder, bilateral seminal vesicles and the distal anterior wall of the rectum Enlarged lymph nodes in the pelvic wall and inguinal region Multiple foci of abnormal signal in the pelvis	Prostate adenocarcinoma	/	Leuprorelin Acetate and Apalutamide
Renal cancer	A 4.2 × 3.5 × 4 cm mass in the middle of the right kidney, with possible multiple retroperitoneal lymph node metastases	Clear cell renal cell carcinoma	Laparoscopic partial nephrectomy	/

## Data Availability

The original contributions presented in this study are included in the article. Further inquiries can be directed to the corresponding authors.
